# Despite Affordability, Low Utilization of Lipid Lowering Drugs in Iran: Trend Analysis and Benchmarking 2005-2016

**DOI:** 10.22037/ijpr.2019.1100695

**Published:** 2019

**Authors:** Saman Zartab, Hadi Abbassian, Nasrin Nassiri Koopaei, Mohammad Hajimolaali

**Affiliations:** a *Department of Pharmacoeconomics and Pharmaceutical Administration, Tehran University of Medical Science, Tehran, Iran.*; b *Department of Food and Drug, Shahid Beheshti University of Medical Sciences, Tehran, Iran.*; c *Department of Drug and Food Control, Faculty of Pharmacy, Students’ Scientific Research Center, Tehran University of Medical Sciences, Tehran, Iran.*

**Keywords:** Dyslipidemia, Drug utilization, Lipid-lowering medications, Affordability, Defined daily dose, ATC codes

## Abstract

Dyslipidemia is responsible for great mortality and morbidity each year. Little data are available on the availability and affordability of Dyslipidemia medications in low and middle incomes countries. In a retrospective time-series study, we examined the utilization pattern and affordability of lipid-lowering medications in Iran as a lower middle-income country. We initially calculated the defined daily dose for 1000 inhabitants (DID) in different years and compared the results with OECD member countries in the same year. We also used 90% Drug Utilization method to rank and compare lipid lowering drugs with the WHO Essential Medicines List (EML). We measured the affordability by the minimum daily wage for one-month course of treatment. The use of lipid-lowering medications increased from 6.31 to 45.98 DID between 2005 and 2016. The utilization share of the subgroup of statins was above 80% of total utilization. Compared to OECD countries, Iran utilized 40% of the average utilization in 2015. In 2015, Atorvastatin was on 90% of DU medications. At the beginning of the study, only Lovastatin and Nicotinic acid were affordable in 2005, but at the end of the study, all lipid-lowering medications were affordable. The utilization of lipid-lowering medications, despite being affordable, was low. One of its possible reasons is the lack of proper management of patients with Dyslipidemia and low adherence of patients. Another possible cause is the high percentage of undiagnosed patients in the community. Therefore, comprehensive planning and policy-making should be taken to increase utilization and eliminate the related obstacles.

## Introduction

Dyslipidemia raises the risk of cardiovascular diseases. Worldwide, high-level cholesterol causes 2.6 million deaths annually and 29.7 million disability-adjusted life years (DALYs). According to WHO estimation, nearly one-third of deaths are related to cardiovascular disease, of which 80% occurs in low- income or middle-income countries (1). There are two main methods for management of dyslipidemia: first, lifestyle modification and second, using lipid-lowering medications. Based on clinical trials, reducing total cholesterol is associated with reduction of cardiovascular diseases risk (2-4). For example, a 10% reduction in serum cholesterol levels in 40-year-old men has led to a 50% reduction in cardiovascular diseases over a 5-year period. Currently, medications play an important role in controlling serum lipids. Therefore, utilization of lipid-lowering medications should be a top priority for health policymakers. 

Previous studies have shown different patterns of using lipid-lowering medications (5, 6). For example, T. Walley *et al*. examined the pattern of prescribing and utilization lipid-lowering medications in European countries between 1997 and 2003 and found that the use of lipid-lowering medications has increased in all countries, which was due to an increase in statins utilization. They also observed differences in utilization between countries (6).

Rasha Khatib *et al*. reported that between 2003 and 2013, cardiovascular medicines including statins were unaffordable: 14% for citizens in high-income countries, 25% for citizens in upper-middle-income countries, 33% for citizens in lower-middle-income countries, and 60% for citizens in low-income countries (7).

Epidemiologic studies indicate a high prevalence of dyslipidemia in Iran as a country with a lower-middle per capita income country. Esteghamati *et al.* reported the prevalence of hypertriglyceridemia and total cholesterol ≥ 200 at 36.4% and 42.9%, respectively, in 2008. Dyslipidemia is more common among urban residents and older people. He reported a higher prevalence of Hypercholesterolemia in women and higher prevalence of hypertriglyceridemia in men (8). Tabataei-Malazy *et al.* reported in a meta-analysis study in 2014 that the prevalence of Hypercholesterolemia (>200) was 41.6% and hypertriglyceridemia was 46.0%. However, there are very few reports on the utilization pattern of lipid-lowering medications for monitoring the dyslipidemia pharmacotherapy in Iran (9).

This study examined the utilization pattern of lipid-lowering medications during the years 2005-2016. Then, this pattern of utilization was compared with available international data. Finally, the affordability of lipid-lowering medications was explored in Iran to explain a part of the utilization pattern.

## Experimental

*Design*

A retrospective time-series study was conducted to evaluate the utilization pattern of lipid-lowering medications and patient’s affordability in Iran (2005-2016). We then compared drug utilization rates with the latest data from 26 OECD countries in 2015.

*National Data Source*

Annual wholesales data was obtained from the Food and Drug Administration of Iran. Data was recorded based on the sale to drugstores across the country, available electronically in different years. In this database, each drug product (specific form and dose) is identified by a generic code and a special code, related to the manufacturer or importer and distributor of the product in the specified year. 

The list of lipid-lowering medications available in the country’s market during 2005 through 2016 was obtained by consulting a specialist and referring to the Iran National Drug List.

The accuracy of the data was analyzed by comparing the generic code, the name of the manufacturer or importer, and the product dose during consecutive years. To calculate the number of DDDs per 1,000 inhabitants per day (DID), the annual population was extracted from Iran′s Statistics Center.

*International data source*

The data on utilization of lipid-lowering medications in 26 countries, member of the Organization for Economic Co-operation and Development (OECD), obtained from its website (http://www.oecd.org) (10).

*Assessment of the utilization pattern in Iran*

The ATC/DDD method was used to standardize the raw data. The ATC/DDD 2013 guideline was taken from the WHO Collaborating Center website for drug statistical method (11). 

In this study, ATC codes of the C_10_ group and its associated DDD were used to standardize lipid-lowering medications. According to WHO definition “The DDD is the assumed average maintenance dose per day for a drug used for its main indication in adults” (11).

The following formula was used to calculate the number of DDDs per 1,000 inhabitants per day (DID) (12, 13):

$\mathrm{DID}=\frac{\mathrm{number DDDs}\times \mathrm{1000}}{\mathrm{number of population}\times \mathrm{365}}$

Annual utilization of lipid-lowering medications was measured based on different levels of ATC codes and utilization growth rate was calculated. 

The annual use of HMG-CoA reductase and other lipid-lowering medications were calculated. Excel 2010 was used to calculate the trend line of utilization over time. We used the Drug Utilization-90% (DU-90) methodology to compare the pattern of C_10_ utilization with the list of essential medicine was published by the WHO (19^th^ edition 2015) (14-16). We identified the ATC code, accounted for 90% of total DDDs in lipid-lowering medications (13).

*Benchmarking utilization patterns*

Utilization data of C_10 _drugs from 26 OECD countries was compared with Iranian similar data in 2015 (10).

OECD member countries with a prevalence of dyslipidemia similar to Iran (41.6%) were selected and the utilization patterns of these countries during 2005-2016 were compared with the utilization pattern in Iran. 

*Assessing the affordability*

The affordability was defined by comparing the number of minimum daily wage that could cover the cost of one month consumption of treatment protocol (17). To calculate the affordability, the minimum daily wage was obtained from the Ministry of Labor database which is announced annually. If the cost of one-month utilization of medication or treatment was less than the daily wage, we considered that drug affordable (13).

Affordability was calculated for each drug separately based on the monthly cost of hypothetical treatment (30DDDs) (13).

## Results

**Figure 1:**
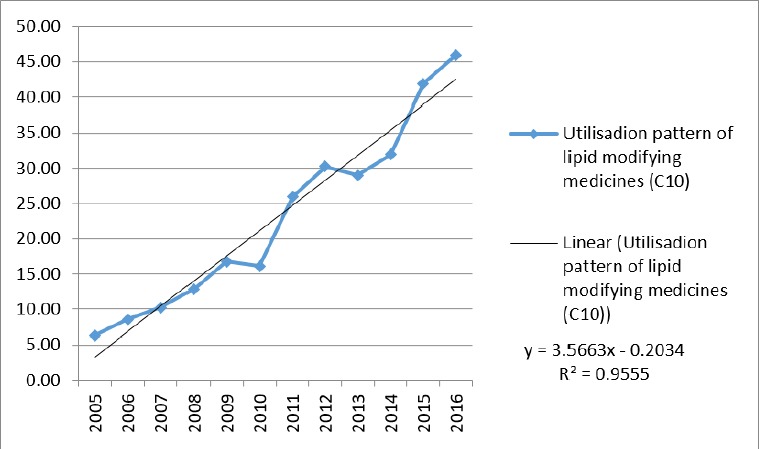
Utilization pattern of lipid lowering agents (C10) in Iran (DDD, defined daily dose)

Use of lipid-lowering medications (C_10_) increased from 6.31 to 45.98 DID during the study period. The annual growth percentage was fluctuating: from 35.0% in 2005 to 9.7% in 2016, while it was negative in 2010 and 2013 (3.5% and 4.0%, respectively). Although the trend in both groups of HMG-CoA reductase and other lipid-lowering medications was positive, the utilization proportion of HMG-CoA reductase was higher than 80% (80.7% in 2005 to 93.5% in 2016). Figure 1 is illustrating the trend of lipid-lowering medications utilization. A linear trend shows a high linear relationship (R^2^ = 0.96).

The overall utilization in the subgroup of HMG-CoA reductase during 2005 to 2016 changed from 5.09 to 43.01 DID. During these 11 years, the growth of this subgroup was relatively stable (R^2^ = 0.96 linear trend). Initially, there were three medications in the market: Atorvastatin (C10AA05), Lovastatin (C10AA02), and Simvastatin (C10AA01), but more than 90% of the HMG-CoA reductase subgroup utilization was associated with Atorvastatin. Lovastatin and Simvastatin had a slow decrease while Atorvastatin rapidly increased (16.8 fold). In 2011, Rosuvastatin entered the market. Until 2015, there was no internal (generic) production of it. Rosuvastatin’s utilization grew very slowly but after domestic production in 2015, its utilization increased drastically, (134 times). Table 1 show a summary of utilization of HMG-CoA reductase subgroup and Table 2 displays the utilization of non HMG-CoA reductase.

**Figure 2:**
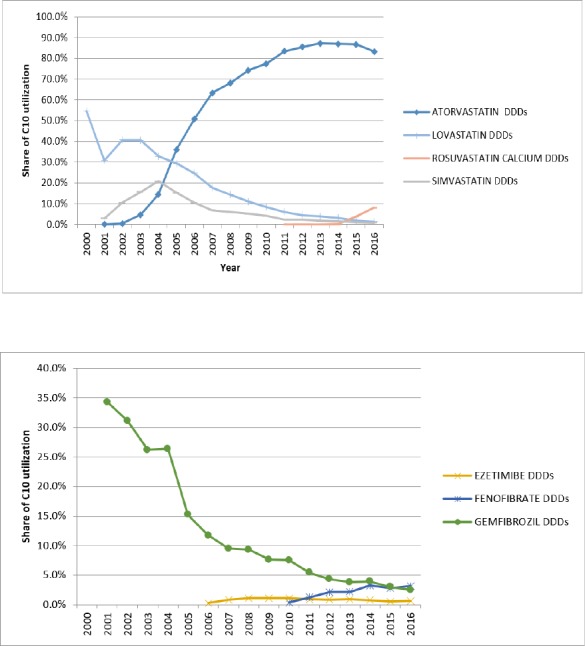
Pattern of DU-90% for lipid lowering drugs in Iran

**Figure 3:**
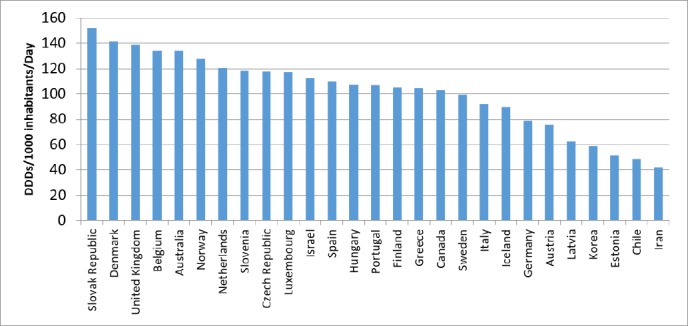
Comparison of lipid lowering drugs utilization in Iran and OECD countries (2015)

**Figure 4:**
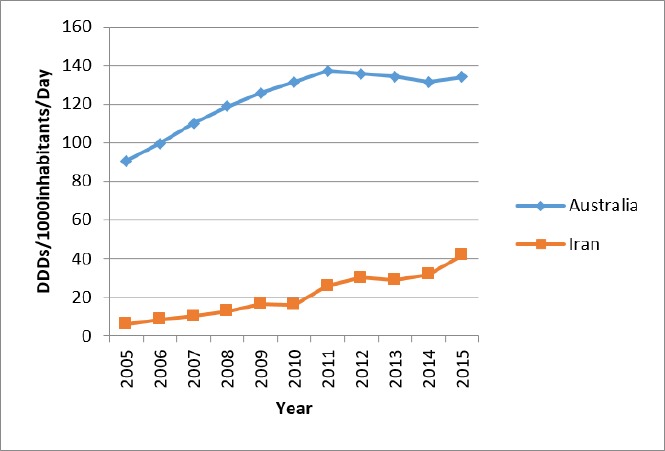
Benchmarking the trend of lipid lowering drugs utilization (Iran and Australia)

**Figure 5:**
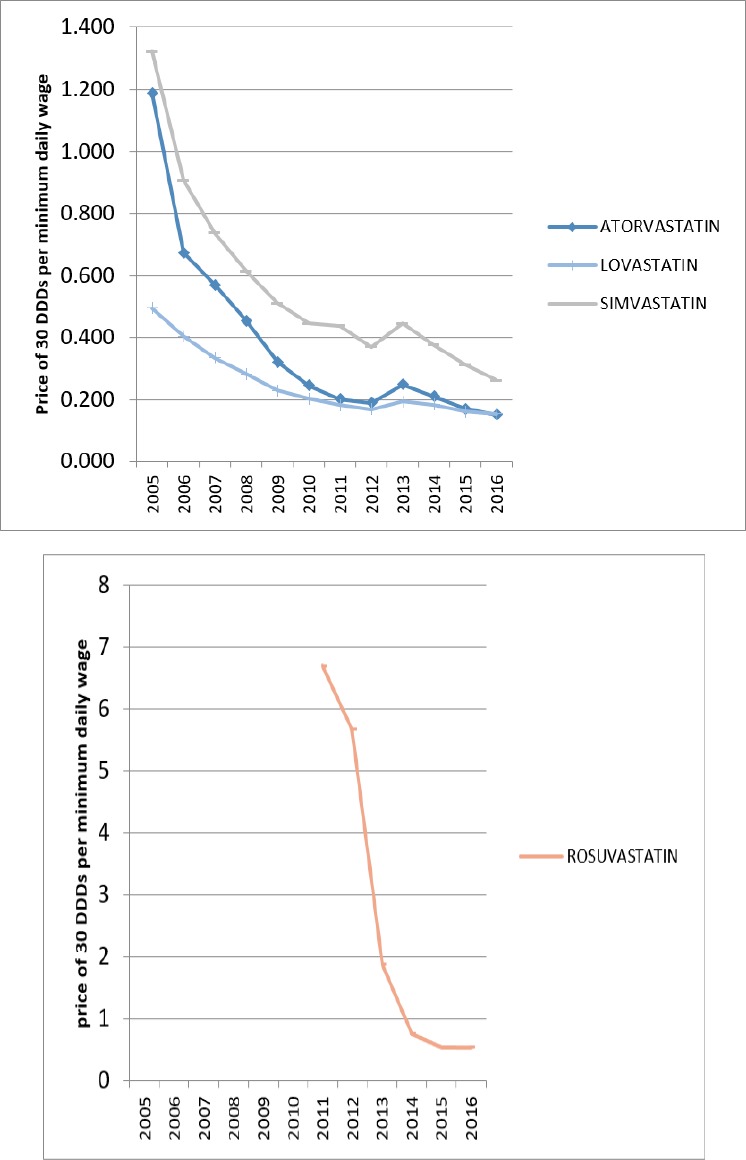
Affordability of statins in Iran

**Figure 6:**
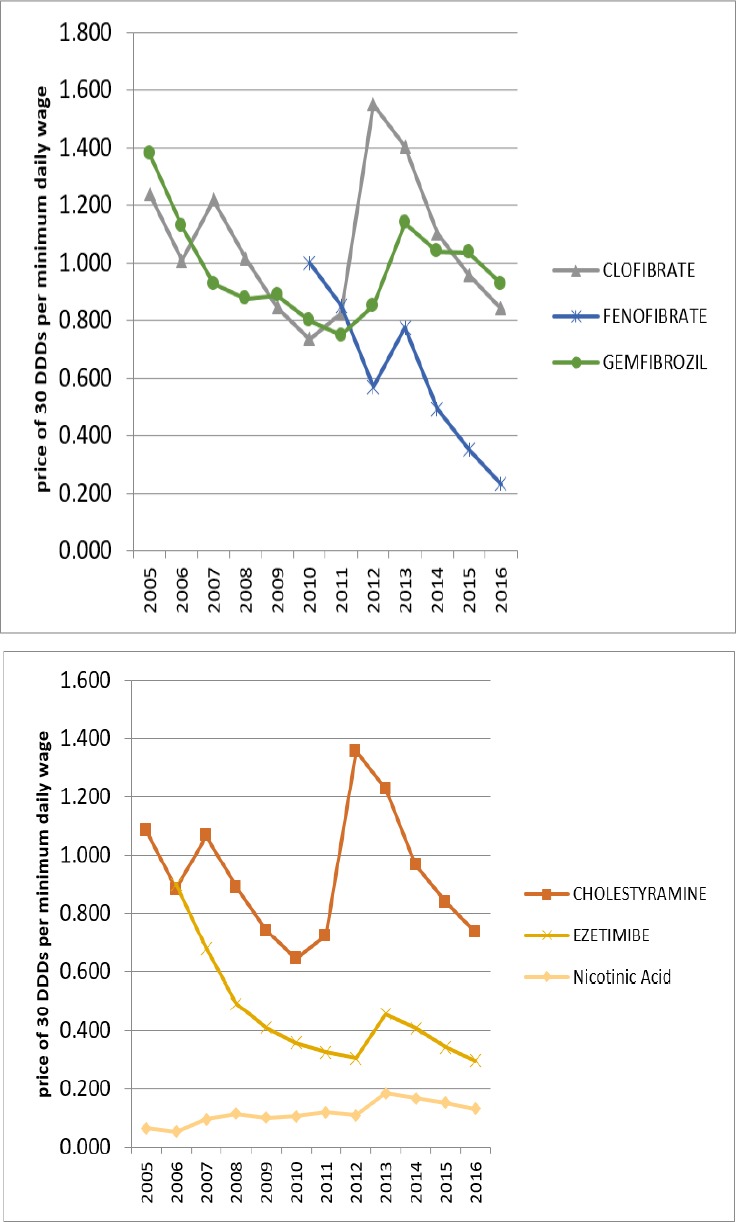
Affordability of non-HMG-CoA reductase

**Figure 7:**
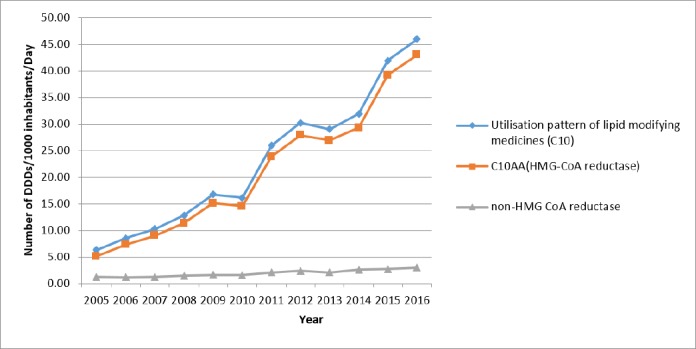
Effect of HMG-CoA reductase utilization on total utilization

**Table 1:** Number of defined daily dose per 1000 inhabitants per day for HMG-CoA reductase

	**2005**	**2006**	**2007**	**2008**	**2009**	**2010**	**2011**	**2012**	**2013**	**2014**	**2015**	**2016**
C10AA05 (Atorvastatin)	2.28	4.32	6.50	8.74	12.41	12.50	21.62	25.86	25.33	27.77	36.32	38.30
C10AA02 (Lovastatin)	1.86	2.11	1.81	1.84	1.85	1.38	1.59	1.36	1.10	1.03	0.77	0.60
C10AA07 (Rosuvastatin)							0.00	0.00	0.00	0.03	1.63	3.71
C10AA01 (Simvastatin)	0.96	0.90	0.71	0.76	0.86	0.68	0.62	0.68	0.51	0.49	0.46	0.39

**Table 2:** Number of defined daily dose per 1000 inhabitants per day for non HMG-CoA reductase

	**2005**	**2006**	**2007**	**2008**	**2009**	**2010**	**2011**	**2012**	**2013**	**2014**	**2015**	**2016**
C10AC01(Cholstyramine)	0.014	0.012	0.010	0.003	0.006	0.006	0.006	0.007	0.006	0.007	0.006	0.006
C10AB10 (Clofibrate)	0.232	0.154	0.152	0.127	0.135	0.117	0.119	0.117	0.085	0.077	0.080	0.076
C10AX09 (Ezetimibe)		0.024	0.085	0.139	0.182	0.173	0.242	0.262	0.255	0.244	0.214	0.278
C10AB05 (Fenofibrate)						0.058	0.316	0.633	0.633	1.029	1.152	1.456
C10AB04 (Gemfibrozile)	0.960	0.997	0.973	1.194	1.278	1.220	1.408	1.308	1.094	1.254	1.266	1.154
C10AD02 (Nicotinic Acid)	0.009	0.008	0.008	0.006	0.008	0.006	0.005	0.002	0.007	0.003	0.002	0.002

During the study, DU-90 medications were identified for HMG-CoA reductase and non-HMG-CoA reductase. At the beginning of the study, Atorvastatin, Gemfibrozil, Lovastatin, and Simvastatin were on the list. By increasing the proportion of Atorvastatin at the end of the study period, only Atorvastatin and Rosuvastatin were on the list (Figure 2). While only simvastatin is on the list of WHO essential medicines (16).

Comparisons with OECD countries showed that Iran utilized less in 2015 (Figure 3). The prevalence of dyslipidemia in Australia was similar to Iran, but the utilization of lipid-lowering medications in Australia was 3.2 times higher than that of Iran in 2015 (Figure 4). Of course, the utilization growth in Australia during 11 years was 1.5 times, while it was 7.3 times in Iran as a developing country.

In 2005, only Lovastatin from HMG-CoA reductase group and Nicotinic Acid from non-HMG-CoA reductase were affordable. But over the years, all of the lipid-lowering medications have moved towards affordability. In 2016, all of the lipid-lowering medications in Iran were affordable. 

The growth of affordability was partly due to the generic plan that can explain some of the utilization growth over these years. Figures 5 and 6 are displaying the affordability of lipid-lowering medications over the years.

## Discussion

Based on the findings of this study, the use of lipid lowering drugs (C_10_) in the 11-year study period (2005-2016) has increased nearly 7 times. There was an increase in utilization of lipid-lowering medications while there was a decrease in the incidence of dyslipidemia over the years (2005-2011). So, in addition to consider the appropriate lifestyle, management of hyperlipidemia has become more efficient in these years.

Compared to OECD countries, the use of C_10_ medications was relatively inadequate in Iran. Average utilization of lipid lowering medications in the OECD countries was 104.3 DID in 2015, while Iranian utilized 41.9 DID in the same year. Maybe one of the most important reasons for this discrepancy is the high number of undiagnosed patients in the community (18).

In 2005, the first year of study, only Lovastatin and Nicotinic acid medications were affordable for Iranians, while in 2016, all Iranian lipid-lowering medications available in the Iranian market were affordable. 

The main reason for affordability of drugs was generic drug pricing policies. Drug prices are extremely under the control of the Ministry of Health of Iran (19).

For many years, Atorvastatin had the highest utilization in the C_10_ group. But after the arrival of the generic form of Rosuvastatin into the market and its affordability, its growth rate was much higher than Atorvastatin growth rate. Part of this phenomenon can be explained by better efficacy of Rosuvastatin (20).

Between 2005 and 2016, the increase in utilization was entirely influenced by the increased use of HMG-CoA reductase subgroup medications. Thus, even total utilization fluctuations have been strongly influenced by the fluctuations in the use of HMG-CoA reductase subgroup medications (Figure 7). 

## Limitations

It should be noted that the effect of insurance coverage was not considered in this study. However, the increased insurance coverage that occurred in these years can partly explain this increase in utilization. The data we received in our study was from the wholesale, and attention should be paid to the interpretation of the data. For example, data from expired medications at the pharmacy or at home were not removed from this data. Data from the OECD countries were provided by the competent authorities of each country. Some countries use wholesale data, while others use prescription data. They maybe differ in collecting non-reimbursement drugs and OTC sales data. However, Denmark, Estonia, Finland, Sweden, and the Slovak Republic used wholesale data for the report, like Iran (21). 

## Conclusion

To summarize, the utilization of lipid-lowering medications has increased in Iran over the past 11 years as a middle-income country, although utilization is still low in comparison to OECD member due to the high rates of undiagnosed patients, inappropriate education on utilization, and inappropriate disease control in diagnosed patients. While all of the lipid-lowering medications in Iran are affordable, for controlling dyslipidemia, comprehensive plans should be provided to educate patients to enhance their adherence and to improve the patients’ lifestyle (22).
